# UL36 Rescues Apoptosis Inhibition and *In vivo* Replication of a Chimeric MCMV Lacking the M36 Gene

**DOI:** 10.3389/fcimb.2017.00312

**Published:** 2017-07-14

**Authors:** M. Zeeshan Chaudhry, Bahram Kasmapour, Carlos Plaza-Sirvent, Milica Bajagic, Rosaely Casalegno Garduño, Lisa Borkner, Tihana Lenac Roviš, Andrea Scrima, Stipan Jonjic, Ingo Schmitz, Luka Cicin-Sain

**Affiliations:** ^1^Department of Vaccinology and Applied Microbiology, Helmholtz Centre for Infection Research Braunschweig, Germany; ^2^German Center for Infection Research Braunschweig, Germany; ^3^Research Group Systems-Oriented Immunology and Inflammation Research, Helmholtz Centre for Infection Research Braunschweig, Germany; ^4^Institute of Molecular and Clinical Immunology, Otto-von-Guericke University Magdeburg, Germany; ^5^Young Investigator Group Structural Biology of Autophagy, Helmholtz Centre for Infection Research Braunschweig, Germany; ^6^Faculty of Medicine, Center for Proteomics, University of Rijeka Rijeka, Croatia; ^7^Department for Histology and Embryology, Faculty of Medicine, University of Rijeka Rijeka, Croatia; ^8^Institute for Virology, Medical School Hannover Hannover, Germany

**Keywords:** cytomegalovirus, apoptosis, UL36, M36, Transgenic virus

## Abstract

Apoptosis is an important defense mechanism mounted by the immune system to control virus replication. Hence, cytomegaloviruses (CMV) evolved and acquired numerous anti-apoptotic genes. The product of the human CMV (HCMV) UL36 gene, pUL36 (also known as vICA), binds to pro-caspase-8, thus inhibiting death-receptor apoptosis and enabling viral replication in differentiated THP-1 cells. *In vivo* studies of the function of HCMV genes are severely limited due to the strict host specificity of cytomegaloviruses, but CMV orthologues that co-evolved with other species allow the experimental study of CMV biology *in vivo*. The mouse CMV (MCMV) homolog of the UL36 gene is called M36, and its protein product (pM36) is a functional homolog of vICA that binds to murine caspase-8 and inhibits its activation. M36-deficient MCMV is severely growth impaired in macrophages and *in vivo*. Here we show that pUL36 binds to the murine pro-caspase-8, and that UL36 expression inhibits death-receptor apoptosis in murine cells and can replace M36 to allow MCMV growth *in vitro* and *in vivo*. We generated a chimeric MCMV expressing the UL36 ORF sequence instead of the M36 one. The newly generated MCMV^UL36^ inhibited apoptosis in macrophage lines RAW 264.7, J774A.1, and IC-21 and its growth was rescued to wild type levels. Similarly, growth was rescued *in vivo* in the liver and spleen, but only partially in the salivary glands of BALB/c and C57BL/6 mice. In conclusion, we determined that an immune-evasive HCMV gene is conserved enough to functionally replace its MCMV counterpart and thus allow its study in an *in vivo* setting. As UL36 and M36 proteins engage the same molecular host target, our newly developed model can facilitate studies of anti-viral compounds targeting pUL36 *in vivo*.

## Introduction

Human cytomegalovirus (HCMV), a member of the betaherpesvirus family, retains global endemic status, with infection rates ranging from 30% in industrialized countries to more than 90% in developing ones (Manicklal et al., 2013). HCMV infection induces mild or no symptoms in immunocompetent people, yet the immunocompromised or immunodeficient ones suffer severe bouts of HCMV-related disease due to virus reactivation or de novo infection. Antivirals like gancyclovir or foscarent, are staple therapy against HCMV, yet all approved drugs act by inhibiting viral DNA polymerization, prompting the selection of resistant mutants and impairing our ability to use them in combination therapy. Therefore, identifying and validating additional molecular targets in HCMV-infected cells remains a clinically important goal.

Due to its strict host specificity, HCMV cannot infect non-human cells. Hence, the study of HCMV biology is limited to *in vitro* models of infection (Kim and Carp, 1972; Lafemina and Hayward, 1988; Tang and Maul, 2006), or to the study of HCMV homologs that coevolved with other animal species. The mouse homolog of HCMV is the mouse cytomegalovirus (MCMV), and it provides a widely used tool to study the CMV biology *in vivo* (Reddehase et al., 2002). However, the study of the function of individual MCMV genes does not necessarily reflect the molecular interaction of orthologous HCMV-encoded gene products and target molecules within human host-cells. Therefore, molecular interactions between MCMV proteins and their ligands may not be extrapolated to define molecular targets of HCMV infection. Similarly, chimeric MCMVs expressing HCMV genes instead of the MCMV orthologue gene showed impaired replication, (Wagner et al., 2000; Farrell et al., 2011) limiting their use as tools to study the function of HCMV genes *in vivo*.

Apoptosis is a complex mechanism that was originally defined as programmed cell death characterized by cell shrinkage, pyknosis, DNA fragmentation and plasma membrane blebbing (Kerr et al., 1972). The two major apoptosis pathways are the mitochondrial and the death-receptor apoptosis, responding to cell-intrinsic or to extrinsic stimuli, respectively (Galluzzi et al., 2012). The intrinsic mitochondrial apoptosis can be triggered by a plethora of stress signals that converge at the level of engagement of proteins of the Bcl2 family in the mitochondrion (Kroemer et al., 2007). The extrinsic apoptosis pathway, on the other hand, is initiated by extracellular ligands that bind to receptors of the tumor necrosis factor (TNF) superfamily. Either pathway leads to the activation of cysteine-aspartic acid proteases called caspases, which mediate a cascade of signals culminating in DNA fragmentation and cell death (Ashkenazi and Dixit, 1998). CD95 (also known as Fas or Apo1) is the best characterized member of the tumor necrosis factor (TNF) receptor gene superfamily (Huttmann et al., 2015). The death receptors undergo homo-trimer formation upon binding of the corresponding death ligand, which recruits the adaptor protein FADD (Fas-associated death domain) (Chinnaiyan et al., 1995), activating caspase-8, and initiating the caspase signaling cascade. Caspase-8 auto-catalytically cleaves itself, which releases it from the death-initiation signaling complex (DISC) into the cytosol, where it acts on downstream effector caspases, like caspase-3, and commits the affected cell to apoptosis (Muzio et al., 1998).

Intrinsic apoptosis is employed as a cell autonomous defense mechanism that restricts the spread of viruses. Similarly, immune cells also induce apoptosis via death receptor signaling (Ashkenazi and Dixit, 1998). Consequently, the ability to inhibit cell death confers survival advantage to the viruses that have evolved antiapoptotic viral genes. Multiple genes disrupting diverse apoptotic signaling pathways (Goldmacher et al., 1999; Brune et al., 2001, 2003; Skaletskaya et al., 2001; Menard et al., 2003) have been identified in the case of cytomegaloviruses. The importance of antiapoptotic genes is highlighted by the remarkable conservation of viral antiapoptotic proteins among different cytomegaloviruses (Mccormick et al., 2003). The HCMV viral Inhibitor of Caspase-8 Activation (vICA) is encoded by the UL36 gene. The mouse cytomegalovirus gene M36 is the positional orthologue of UL36 and it has 23% amino acid identity to UL36 (Skaletskaya et al., 2001; Menard et al., 2003). Both of these genes encode proteins that inhibit death receptor mediated apoptosis by binding to caspase-8 and inhibiting its activation (Skaletskaya et al., 2001; Menard et al., 2003; Cicin-Sain et al., 2008). MCMV mutants lacking M36 (ΔM36.MCMV) show growth defects in cultured macrophages, which undergo apoptosis upon infection (Menard et al., 2003; Cicin-Sain et al., 2008). Similarly, the UL36 gene is required for HCMV growth in THP-1 derived macrophages, but it inhibited both the apoptotic, as well as the non-apoptotic cell death pathways (Mccormick et al., 2010). We have previously identified the M36 gene as a determinant of viral fitness *in vivo* and shown that this fitness and apoptosis inhibition are restored in a ΔM36 MCMV by the expression of a dominant-negative FADD gene (Cicin-Sain et al., 2008). More recently, it was shown that the molluscum contagiosum gene MC159 may also replace the M36 function and protect the virus from apoptosis, and improve its replication fitness in macrophages, albeit not completely (Huttmann et al., 2015). We also showed that macrophages are major immune system cells that control the growth of ΔM36.MCMV (Ebermann et al., 2012). The sequence and functional homology of M36 and UL36 may advocate a possible *in vivo* protective function of the UL36 gene, but this could not be confirmed, due to the aforementioned strict HCMV host specificity.

Here we show that the UL36 gene can inhibit death receptor mediated apoptosis in murine cells. We generated a chimeric MCMV virus, where the M36 open reading frame (ORF) was replaced with the UL36 ORF. We demonstrated that UL36 replaces the function of M36 in MCMV infection. While ΔM36.MCMV showed a severe loss of fitness in macrophages and *in vivo*, the expression of UL36 rescued the viral growth. Furthermore, UL36 was bound to caspase-8 in murine cells and blocked apoptosis. Taken together, our data indicate that UL36 and M36 share sufficient functional homologies to enable them to engage the highly conserved cellular target proteins across host species.

## Materials and methods

### Mouse strains

All animal experiments were conducted according to guidelines defined by the Federation of Laboratory Animal Science Associations and the national animal welfare body *Gesellschaft für Versuchstierkunde/Society of Laboratory Animals*. C57BL/6 and BALB/c mice were purchased from Janvier (Le Genest St Isle, France). The experimental procedures were approved by the responsible state office (Lower Saxony State Office of Consumer Protection and Food Safety) under permit no. 33.19-42502-04-12/0838.

### Cells and viruses

HEK293, IC-21, J774A.1, M2-10B4, NIH3T3, and RAW 264.7 cells were purchased from American Type Culture Collection. HEK293, J774A.1, M2-10B4, NIH3T3, and RAW 264.7 cells were maintained in Dulbecco's modified Eagle's medium (DMEM) with supplements (10% FCS, 2 mM L-glutamine, 100 IU/mL penicillin and 100 μg/mL streptomycin). IC-21 cells were maintained in RPMI-1640 medium with above stated supplements. The primary C57BL/6 mouse embryo fibroblasts (MEFs) were prepared and maintained as described earlier (Podlech et al., 2002). The wild type virus MCMV^WT^ is pSM3fr-MCK-2fl derived from Smith strain (Jordan et al., 2011). The recombinant MCMV^UL36^ and ΔM36.MCMV were generated with lambda red-mediated markerless recombination (Tischer et al., 2010; Jordan et al., 2011). The ΔM36.MCMV virus was generated by replacing the start codon and methionine codon in exon 1 of M36 ORF, at position 49,270 and 49,090 respectively, with stop codons. The MCMV^UL36^ virus was generated by replacing the M36 gene in MCMV genome with the UL36 gene in a manner that M36 exons were replaced with UL36 exons in two successive recombination rounds while the M36 intron was left unchanged. The UL36 ORF exons were amplified from HCMV strain Merlin (Dolan et al., 2004). Briefly, the *En Passant* insertion cassettes were generated by cloning UL36 exons around a kanamycin resistance gene and a I-SceI recognition site, which were then amplified using homology primers corresponding to the insertion site in the MCMV genome. This linear DNA was transformed in the electrocompetent and recombination competent GS1783 bacteria harboring MCMV BAC. The bacteria were spread on kanamycin supplemented LB agar plates to select the positive clones and incubated overnight at 32°C. On the following day, the colonies were selected and screened with colony PCR and the sequence was confirmed by Sanger sequencing of the target region. The viruses were recovered by transfecting C57BL/6 MEFs with virus BAC using FuGENE® HD (Promega, CA, USA). Viruses were propagated on M2-10B4 cells and virus stocks were prepared by purification using sucrose cushion as described previously (Menard et al., 2003; Jordan et al., 2011). The virus titers were quantified by plaque assay as PFU/mL using C57BL/6 MEFs.

### Illumina sequencing

The MCMV^WT^ and MCMV^UL36^ BAC DNA was sheared by sonication and the NEBNext® Ultra kit was used for the DNA library preparation. The sequencing was performed on Illumina MiSeq platform. The whole genome DNA sequencing data was analyzed by alignment to reference genome using Burrows-Wheeler Aligner MEM algorithm (Li and Durbin, 2009). The mutations were called using samtools (Li et al., 2009) and VarScan 2 (Koboldt et al., 2012). The parameters used to call mutations (both SNPs and Indels) from aligned sequence were: minimum basecall quality (>20), read depth (≥20) and variant frequency threshold (>1%).

### Plasmids and transfection

Cloning was performed using mammalian expression vector pEBFP-N1, where EBFP gene was deleted and mNeon and N-terminus myc tagged UL36 were inserted using seamless cloning as described earlier (Gibson et al., 2009). The newly developed vector was named mNeon-P2A-mycTAG-UL36-N1 and empty vector was mNeon-N1. HEK293 and NIH3T3 cells were transfected using FuGENE® HD reagent (Promega, CA, USA) according to manufacturer's guidelines.

### *In vitro* infection and virus quantification

Cells were seeded at a defined density one day before infection. On the day of infection, the cells were infected at the required MOI. After 1 h the virus was removed and the cells were washed with 1X PBS and then given fresh growth medium. The growth curves in IC-21, J774A.1, and RAW 264.7 cells were determined by seeding 50,000 cells per well in 24 well plates. The cells were infected the following day, with an MOI of 1 for IC-21 and J774A.1 and an MOI of 2.5 for RAW 264.7. The supernatants were collected on 6 consecutive days and titrated on C57BL/6 MEFs as described previously (Cicin-Sain et al., 2005).

### *In vivo* infection and virus quantification

Six to eight week-old BALB/c and C57BL/6 mice were inoculated with 2 × 10^5^ and 10^6^ PFU, respectively, via the intraperitoneal route, of cell culture-grown and sucrose cushion-purified virus. The mice were housed in specific pathogen free conditions and sacrificed by CO_2_ asphyxiation on day 5 or day 21. Spleen, lungs, liver and salivary glands were collected aseptically and stored in 1 mL medium (DMEM supplemented with 5% FCS, 2 mM glutamine, 100 IU/mL penicillin and 100 μg/mL streptomycin) at −80°C. Organs were homogenized in 5 ml of DMEM (supplemented with 5% FCS, 2 mM glutamine, 100 IU/mL penicillin and 100 μg/mL streptomycin) on 100 μm-pore-size cell strainers. Organ homogenates were used for titration on C57BL/6 MEFs as described above.

### Reverse transcription polymerase chain reaction (RT-PCR)

NIH3T3 cells were infected with MCMV^WT^ and MCMV^UL36^ at an MOI of 1.0. The mRNA was isolated with Magnetic mRNA isolation kit (New England BioLabs) according to manufacturer's instructions. DNase I treated mRNA was used to produce the cDNA by SuperScript® II Reverse Transcriptase (Thermo Fisher Scientific) using the oligo dT primer. Reverse transcription PCR was performed with UL36Exon2-seq-F2 (5′-TTTTTTGGGATGTTGACAGGTG-3′) and UL36Exon2-seq-R3 (5′-GCCCACCATGAAGGATTTTC-3′) primers yielding a 604 bp product.

### Apoptosis assay

Apoptosis was induced in HEK293 and NIH3T3 cells by exposing the cells to 100 ng/mL CD95L and 10 μg/mL cycloheximide (CHX). Active caspase-3 was stained with either FITC or PE labeled anti-active caspase-3 antibodies (BD-Clone C92-605), following CD95L induced apoptosis in HEK293 and NIH3T3, or virus induced apoptosis in murine macrophage cell lines. The protocol used for active caspase-3 staining was adopted from Cicin-Sain et al. (2008). Briefly, cells were gently scraped and pelleted by spinning at 500 g for 5 min and then washed once with PBS. The cells were then re-suspended in 450 μL PBS and fixed by adding 50 μL fixation buffer (eBioscience). This was followed by autofluorescence quenching in 50 mM NH_4_Cl-PBS (500 μL) for 30 min at room temperature. Cells were then stained by suspending them in 100 μL permeabilization solution (eBioscience) and 20 μL antibody solution, and incubated for 1 h in darkness at room temperature. After staining, the cells were washed once and then suspended in FACS buffer. The cells were then acquired in a flow cytometer and analyzed with FlowJo software.

For annexin V staining the cells were harvested as described in previous paragraph. After washing, cells were suspended in 250 μL 1X Annexin V binding buffer (BD, NJ, USA) containing 5 μL of APC labeled Annexin V (BD, NJ, USA). The cells were incubated at room temperature for 30 min and acquired directly in Annexin V binding buffer by flow cytometer.

### Co-immunoprecipitation (Co-IP)

For co-immunoprecipitation of endogenous caspase-8 with UL36, NIH3T3 cells were transfected with myc-tagged UL-36. Cells were harvested 48 h post transfection and lysed in TPNE buffer (buffer 1% v/v Triton X-100, 300 mM NaCl, 8.1 mM Na_2_HPO_4_, 2.7 mM KCl, 1.5 mM K_2_HPO_4_, 2 mM EDTA, 10 mM NaF, 1 mM PMSF, 5 μg/ml apotinin, 5 μg/ml leupeptin, 5 μg/ml pepstatin A, 5 μg/ml chymostatin, 1 mM Na_3_VO_4_, pH7.4). Extracts were incubated overnight at 4°C with 2 μg of anti-myc antibody (#9402, Cell Signaling Technology) in the presence of magnetic Protein G beads (Thermo Fisher Scientific). Subsequently, precipitates were washed, denatured and eluted according to the manufacturer's instructions. Proteins were separated on a 12% polyacrylamide gel. For further analysis, the proteins were blotted onto a polyvinylidene difluoride membrane (GE Healthcare), probed with antibodies against caspase-8 (1G12; Enzo), α tubulin (DM1A, Thermo Fisher Scientific) and UL36 (Center for Proteomics, University of Rijeka, Croatia) and developed with ECL Select chemiluminescence reagent (GE Healthcare).

## Results

### UL36 inhibits apoptosis in murine cells

**Figure 1:**
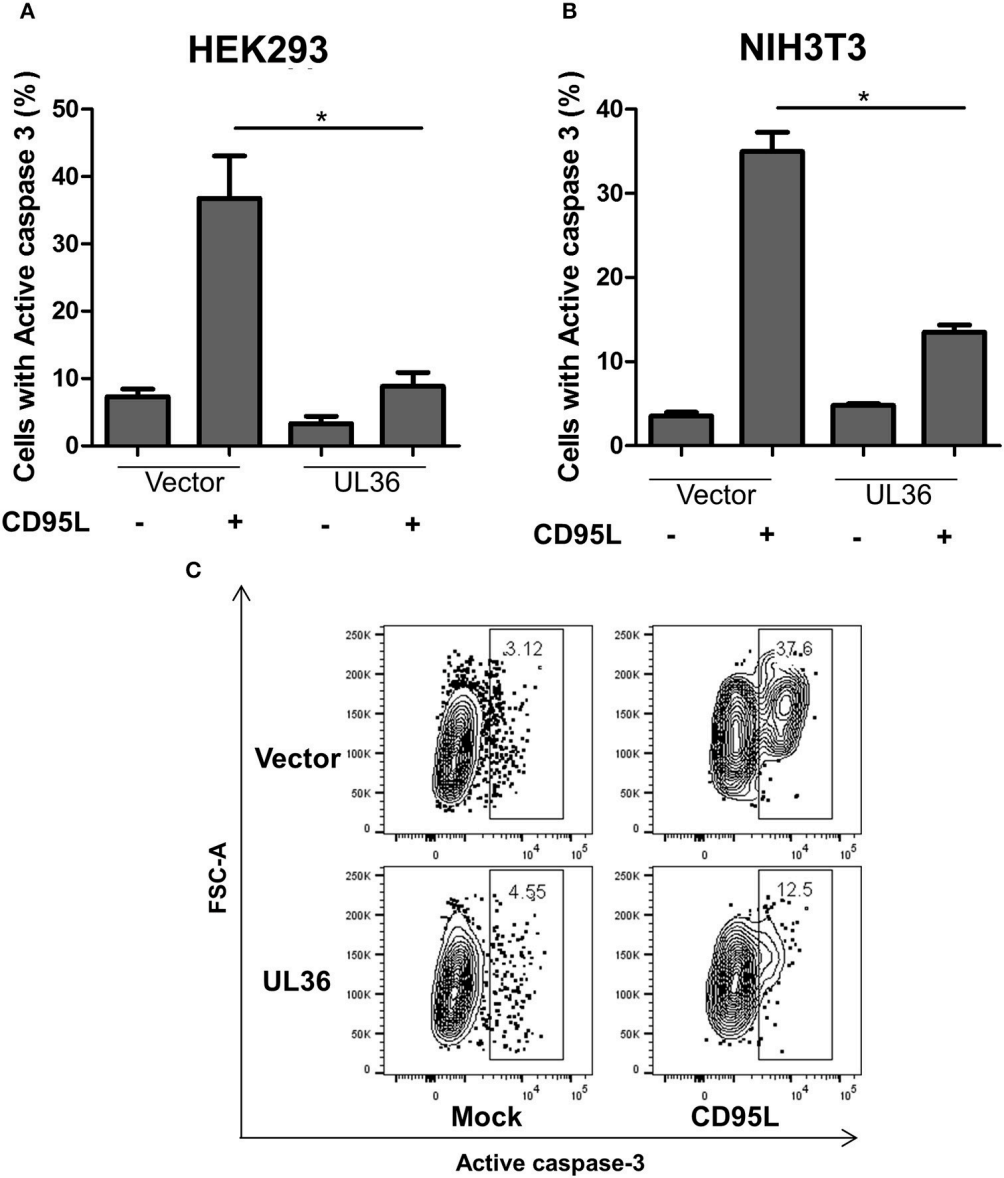
UL36 inhibits apoptosis in Murine NIH3T3 cells. HEK293 **(A)** and NIH3T3 **(B)** cells were transfected with UL36 expressing vector and empty vector. Apoptosis was induced with CD95L 24 h post transfection and the cells were labeled with PE coupled anti-active caspase-3 1 day after stimulation with CD95L, this was followed by flow cytometric analysis. (^*^p < 0.05, Kruskall-Wallis test with Dunns post-analysis). **(C)** Representative contour plots of transfected cells pre-gated on mNeon. The gate shows the percentage of cells with active caspase-3.

A caspase-3 activation assay was used to evaluate if UL36 can inhibit apoptosis in murine cells. NIH3T3 murine cells were transiently transfected with a plasmid expressing UL36, and apoptosis was induced by treating the cells with recombinant CD95L. As control, we transfected the human cell line HEK293 with the same UL36 vector to compare the extent of apoptosis inhibition in human and murine cells. Apoptosis was measured by intracellular staining with fluorophore-coupled antibodies against the active caspase-3, followed by flow-cytometry. Approximately 40–50% of the mock-transfected HEK293 cells showed apoptosis, but UL36-transfection substantially improved viability (Figure 1A). UL36 also inhibited apoptosis in the murine NIH3T3 cells, where a threefold reduction was observed, similar to the one observed in HEK293 cells (Figure 1).

### Generation and characterization of the recombinant MCMV^UL36^

**Figure 2:**
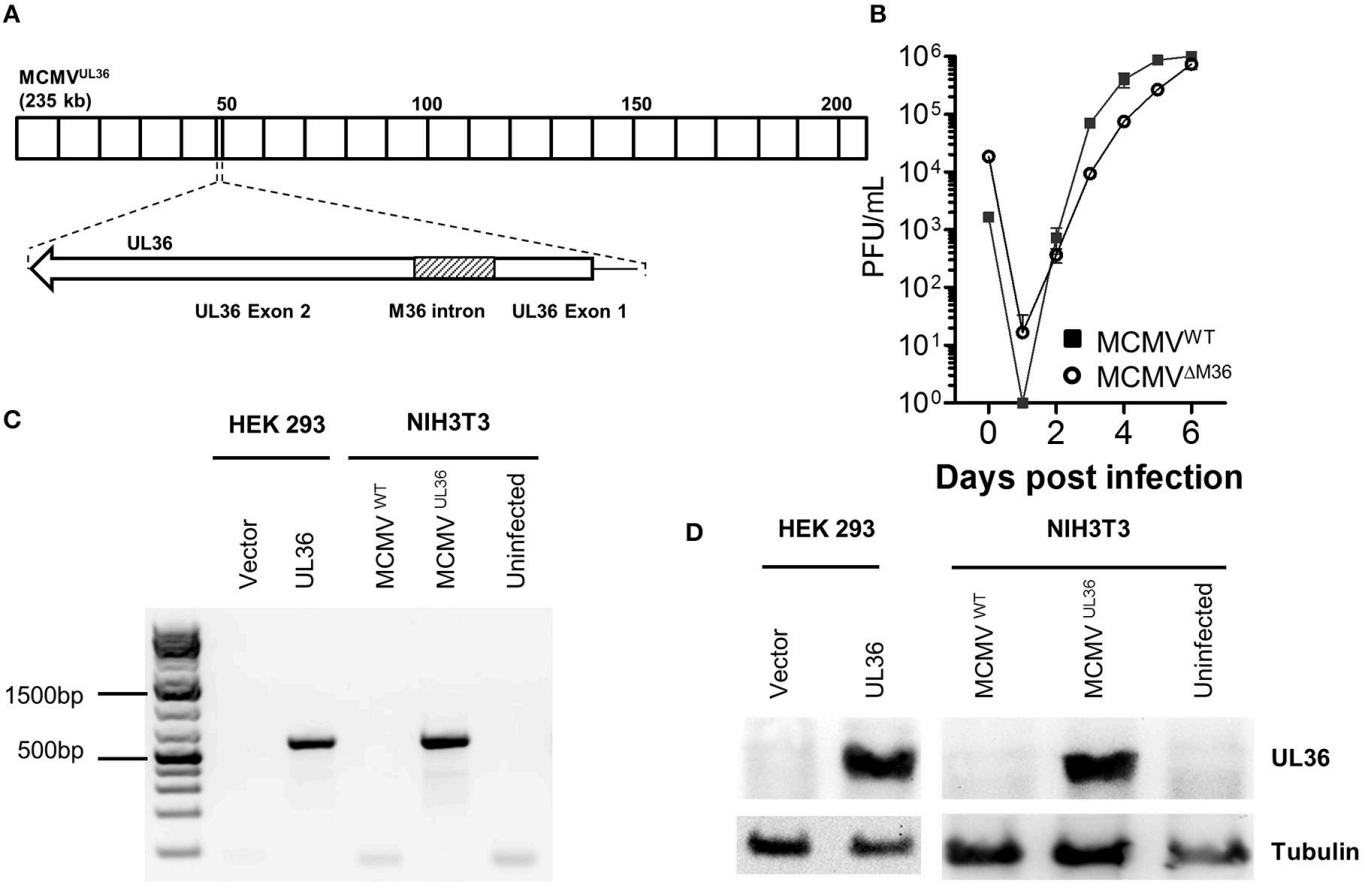
Recombinant MCMV^UL36^ generation and characterization. **(A)** The MCMV M36 ORF (around ~47 to ~49 kbp) was replaced with the UL36 ORF in the MCMV genome by homologous *En passant* recombination. Note that M36 intron was left unchanged in the recombinant MCMV^UL36^ genome. **(B)** NIH3T3 cells were infected with indicated viruses at an MOI of 0.1. The supernatants were collected at indicated days post infection and titrated on C57BL/6 MEFs. Values show the mean of three biological replicates and error bars represent SEM. **(C)** Reverse transcription PCR was performed to detect UL36 mRNA in lysates of HEK293 cells transfected with mNeon-P2A-mycTAG-UL36-N1 or the parental vector or NIH3T3 cells infected with MCMVWT and MCMVUL36 at MOI of 1. **(D)** Western blot for pUL36 in the cell lysate of cells transfected or infected as in **(C)**.

To examine if UL36 can functionally replace M36 and inhibit apoptosis in the context of virus infection, the MCMV^UL36^ virus was generated. M36 and UL36 gene have two exons, where the first exon is smaller and the second one is comparatively larger (Skaletskaya et al., 2001; Cicin-Sain et al., 2005). The intron of the HCMV gene UL36 encodes an miRNA called miRUL36 that inhibits apoptosis in human cells (Guo et al., 2015). Thus, to exclude that any anti-apoptotic activity in the chimeric virus would be due to changes in the non-coding introns, rather than in the exons, MCMV^UL36^ was generated by replacing M36 ORF with that of UL36 in a way that exons of the M36 gene were replaced with UL36 exons and the M36 intron with splice donor and acceptor sites was not altered (Figure 2A). The chimeric virus was generated using BAC-based two-step traceless homology recombination and the whole sequence of the recombinant BAC was analyzed by Illumina next generation sequencing (NGS), where no unwanted mutations, either in the target region or off-target, were found (data not shown). The virus was recovered by transfecting NIH3T3 cells with recombinant BAC and the virus fitness was initially tested *in vitro* by multistep growth curve in fibroblasts. NIH3T3 fibroblast cells were infected with the recombinant virus and its growth kinetics were compared with that of wild-type virus. The growth pattern of both viruses was very similar, arguing for no fitness loss in cultured fibroblasts (Figure 2B), which was in line with the lack of off-target mutations observed by NGS. RT-PCR and western blot were used to confirm the gene expression of the UL36 from the MCMV^UL36^. Since the UL36 gene was inserted under the control of the M36 promotor, its transcription and translation was anticipated to reflect the pattern of M36 expression. UL36 expression was validated on day 1 post infection in NIH3T3 cells infected with MCMV^UL36^ at an MOI of 1.0. The transcription of the gene was verified by analyzing the mRNA of infected cells by RT-PCR with primers specific for UL36 exon 2 (Figure 2C). Protein expression of UL36 by the recombinant virus was confirmed with western blot as shown in Figure 2D, using anti-UL36 monoclonal antibodies that detected the 52 KDa pUL36 protein band. No signal was detected in the ΔM36.MCMV or MCMV^WT^ infected cells (Figure 2D), confirming that the antibodies specifically detected pUL36 and did not cross-react with the M36 protein.

### UL36 expression rescues ΔM36.MCMV replication *in vivo*

We have previously shown that M36 deletion mutants have severe growth defects *in vivo* (Cicin-Sain et al., 2005), which can be rescued by another gene that inhibits death-receptor apoptosis (Cicin-Sain et al., 2008; Ebermann et al., 2012). To confirm that this was indeed due to the lack of the M36 protein (pM36), and not to cis effects, we generated a novel mutant, where the M36 start codon was swapped with a stop codon (see Supplementary Figure 1). Whole viral genome sequence was validated by NGS, and no additional mutations were observed (data not shown). This mutant was used throughout the manuscript and is indicated as ΔM36.MCMV. To evaluate if UL36 can rescue the *in vivo* replication of the MCMV that lacks the M36 protein, MCMV^UL36^ was used to infect BALB/c and C57BL/6 and the growth of the virus was quantified in different organs and compared with the MCMV^WT^ and ΔM36.MCMV in two independent experiments.

**Figure 3:**
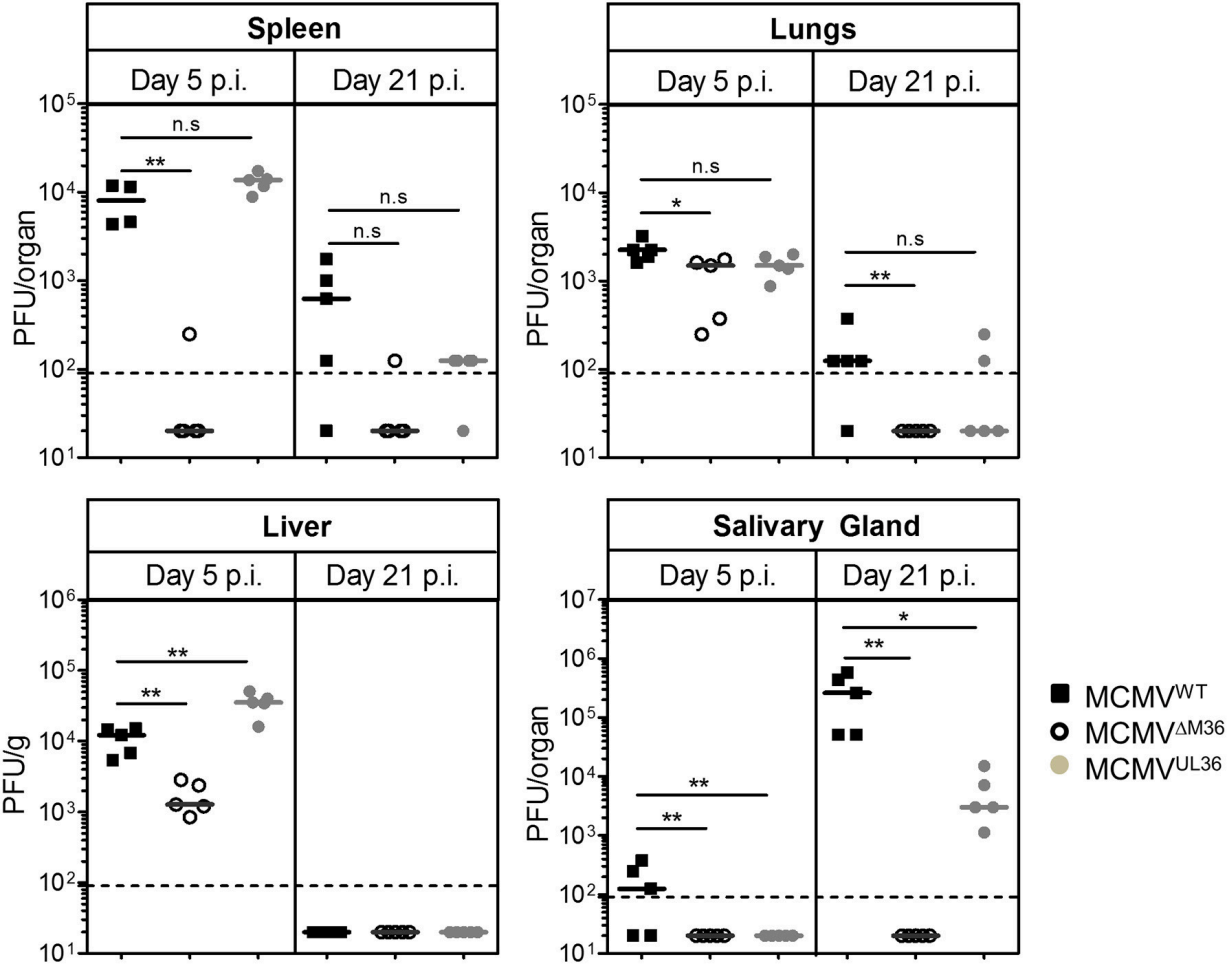
UL36 rescues growth of ΔM36 MCMV in BALB/c mice. BALB/c mice were intraperitoneally infected with 2 × 10^5^ PFUs of indicated viruses. Virus titers in liver, lungs, spleen and salivary gland were determined by organ titration at 5 and 21 day post infection. Each symbol indicates one mice and the solid line represents the median value. The dashed line represents the detection limit for organ titration. (ns – *p* > 0.05, ^*^p < 0.05, ^**^p < 0.01, Mann Whitney test).

BALB/c mice were infected via the intraperitoneal (i.p.) route with 2 × 10^5^ PFU of MCMV^WT^, ΔM36.MCMV or MCMV^UL36^ and organ homogenates were assayed for infectious virus by plaque assay on days 5 and 21 post-infection (Figure 3). At day five post infection, the M36 deletion mutant showed a complete absence of growth in the spleen, lower virus titer in the liver and no loss of titer in the lungs (Figure 3). The MCMV^UL36^ virus, on the other hand, was able to spread to nearby organs from the site of administration and establish virus titers similar to the wild-type virus, indicating that the UL36 gene expression compensated for the absence of the M36 gene and rescued MCMV growth in spleen and liver at day five post infection. On day 21 post infection, virus titers decreased in all organs except the salivary glands, where titers were considerably higher (Figure 3). MCMV^WT^ showed the highest titers in this organ, while ΔM36.MCMV was not detected. MCMV^UL36^ replication in the salivary glands was detected in all infected animals, although the titers were lower than upon MCMV^WT^ infection. Thus, the expression of the UL36 gene helped the mutant virus spread from the site of infection to distant organs and partially rescued the viral growth in the salivary glands.

**Figure 4:**
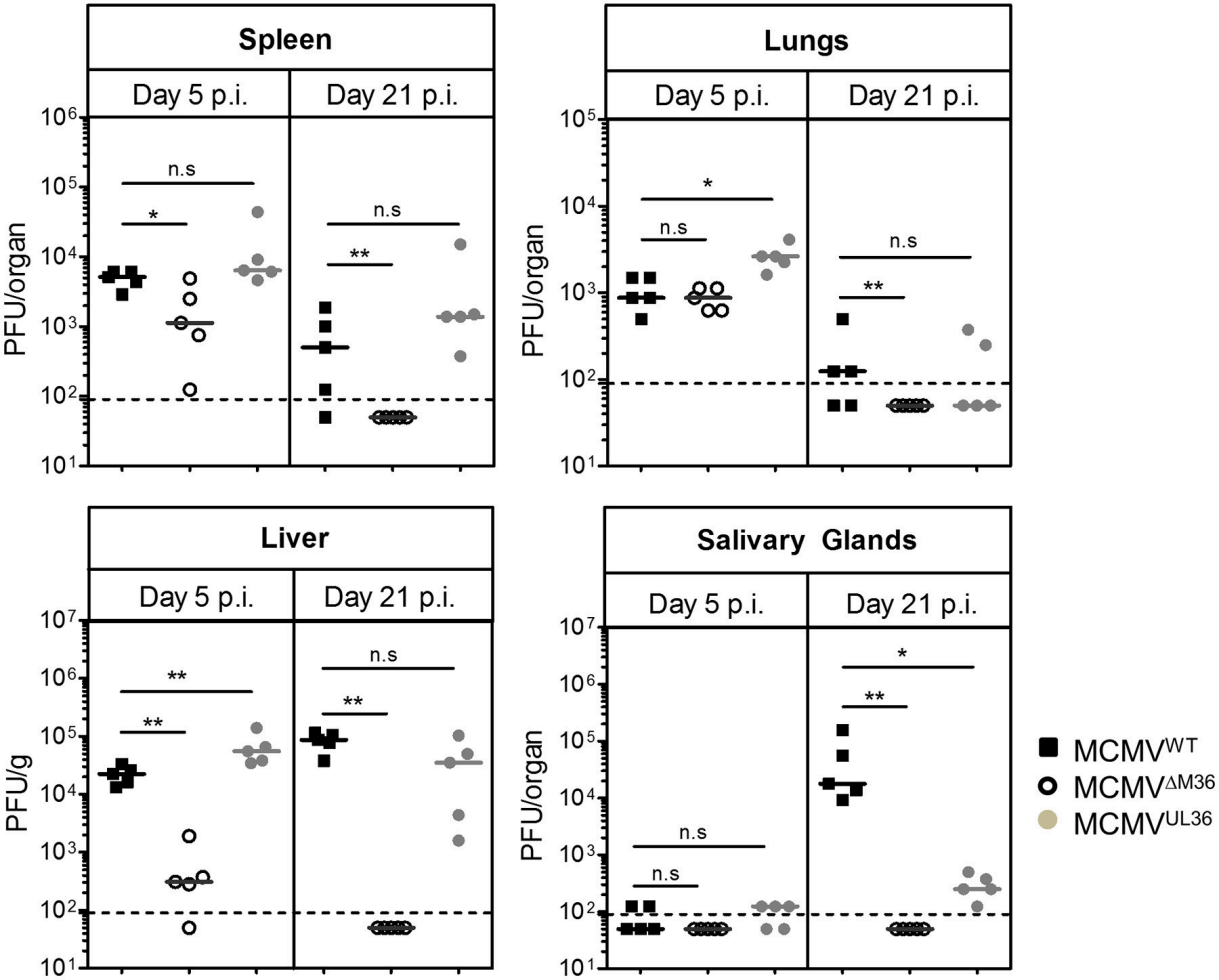
UL36 rescues the growth of ΔM36 MCMV in C57BL/6 mice. C57BL/6 mice were infected with 10^6^ PFU of indicated viruses via the intraperitoneal route. The virus titers in liver, lungs, spleen and salivary gland were determined by organ titration at 5 and 21 day post infection. Each symbol indicates one mice and the solid line represents the median value. The dashed line represents the detection limit for organ titration. (ns – *p* > 0.05, ^*^p < 0.05, ^**^p < 0.01, Mann Whitney test).

To exclude the possibility that UL36-mediated rescue of virus growth is limited to a single inbred mouse strain, we repeated our experiment in C57BL/6 mice. Due to the inherent resistance of C57BL/6 mice against MCMV infection (Brown et al., 2001), they were i.p. infected with a higher infectious dose (10^6^ PFU). As in the BALB/c strain, ΔM36.MCMV did not show severe growth defect in the lungs of the C57BL/6 mice on day five post infection (Figure 4). However, the virus growth was attenuated in the liver and spleen. Viral growth was completely rescued by UL36 expression on day five post infection. In contrast to BALB/c mice, titers of MCMV^WT^ and MCMV^UL36^ were relatively high in the liver and spleen of C57BL/6 mice on day 21 post infection, but ΔM36.MCMV was not detected in any organ on day 21 post infection (Figure 4). Replication of MCMV^UL36^ was also partially rescued in the salivary glands, similar to the rescue observed in BALB/c mice.

### UL36 inhibits apoptosis and rescues ΔM36.MCMV growth in murine macrophages

ΔM36.MCMV is not only growth deficient *in vivo*, but also *in vitro* in macrophage cell cultures (Menard et al., 2003), or in those where macrophages are present (Ebermann et al., 2012). Concomitantly, macrophage cultures infected with M36-deficient MCMV underwent massive apoptosis (Menard et al., 2003), and apoptosis inhibition rescued its growth in these cells (Cicin-Sain et al., 2008). Therefore, it was plausible that MCMV^UL36^ *in vivo* growth would be reflected in its ability to inhibit apoptosis and growth in macrophage cell cultures.

**Figure 5:**
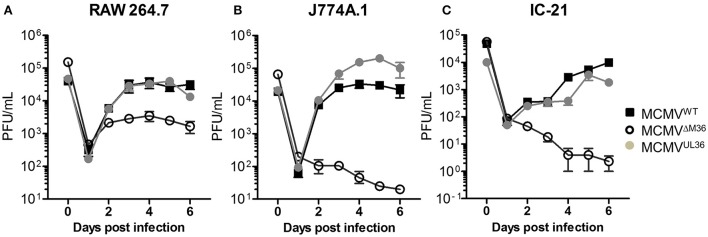
UL36 protein rescues growth of ΔM36 MCMV in macrophages. Growth of MCMV^WT^, ΔM36.MCMV and MCMV^UL36^ was compared in **(A)** RAW 264.7, **(B)** J774A.1, and **(C)** IC-21 macrophage cell lines. RAW 264.7 cells were infected at an MOI of 2.5 while J774.A and IC-21 were infected at 1 MOI. The infectious virus in supernatants was quantified plaque assay on C57BL/6 MEF cells. Values represent means of three biological replicates, while error bars indicate SEM.

The growth of MCMV^UL36^ was compared with MCMV^WT^ and ΔM36.MCMV in three different murine macrophage lines: RAW264.7, J774A.1, and IC-21. J774A.1 and IC-21 cell lines were infected at an MOI of 1 and RAW264.7 cell line was infected with 2.5 PFU per cell, due to its low permissiveness for MCMV infection (data not shown). The ΔM36.MCMV showed growth defects in all macrophage lines (Figure 5). The difference in the growth rate of wild-type MCMV and ΔM36.MCMV was smaller in RAW264.7 than in the other two cell lines (Figure 5A). In RAW264.7 cells, ΔM36.MCMV virus showed some growth during early days of infection, but less than either MCMV^WT^ or MCMV^UL36^. The ΔM36.MCMV did not grow at all in J774.1 (Figure 5B) and IC-21 cells (Figure 5C), as titers continuously decreased in these macrophage cell lines (Figures 5B,C). The expression of the UL36 gene rescued the virus growth, and MCMV^UL36^ growth kinetics were comparable to MCMV^WT^ in all three macrophage cell lines tested.

**Figure 6:**
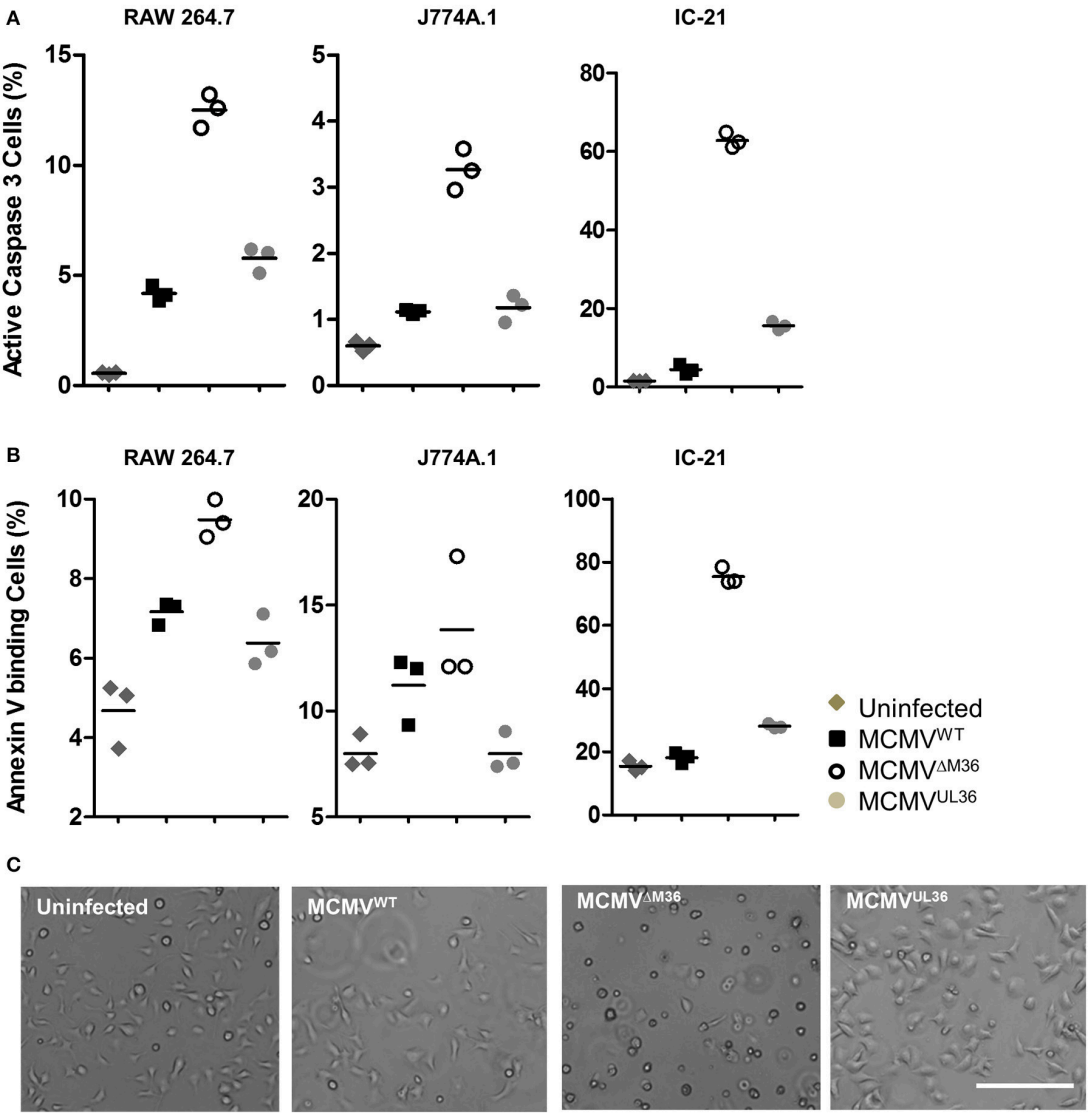
Virus induced apoptosis in macrophages. The indicated macrophages were infected with specified viruses at an MOI of 1 and apoptosis was quantified 1 day post infection. Most experiments were performed at least twice and typical data are shown. **(A)** Active caspase 3 was quantified by antibody labeling and cytofluorometric analysis to asses apoptosis induced by virus infection. **(B)** The plasma membrane eversion of phosphatidylserine was detected by staining with fluorescently labeled Annexin V. Indicated points and horizontal lines on graph represent independent biological replicates and mean values respectively. **(C)** IC-21 cells were infected with indicated viruses at an MOI of 1 and analyzed 24 h post infection. Bar 200 μm.

The growth defects of M36-deficient virus in murine macrophages are due to apoptosis upon infection (Menard et al., 2003; Cicin-Sain et al., 2008). Therefore, it was reasonable to assume that the rescue of growth by UL36 expression would be linked to its ability to inhibit apoptosis in the context of MCMV infection. Apoptosis in MCMV-infected macrophages was quantified by analyzing caspase-3 activation or cell surface expression of phosphatidylserine by flow cytometry. Macrophages were infected with MCMV^UL36^, ΔM36.MCMV, or MCMV^WT^ at an MOI of 1 and apoptosis assays were performed 24 h later. IC-21 cells infected with ΔM36.MCMV showed the highest level of apoptosis in both assays, with more than 60% of cells positive for apoptosis markers (Figure 6), whereas, the percentages of apoptotic cells in the virus-infected RAW 264.7 and J774A.1 cell lines were much lower. UL36 clearly inhibited the induction of apoptosis in the macrophages, since MCMV^UL36^ infection resulted in a percentage of apoptotic cells that was similar to the one observed upon wild-type MCMV infection. Taken collectively, the data argued that the expression of the UL36 gene in the mutant virus lacking the M36 gene inhibits apoptosis and rescues viral growth *in vitro* and *in vivo* by engaging the very same molecular target.

### pUL36 forms complex with murine pro-caspase-8

**Figure 7:**
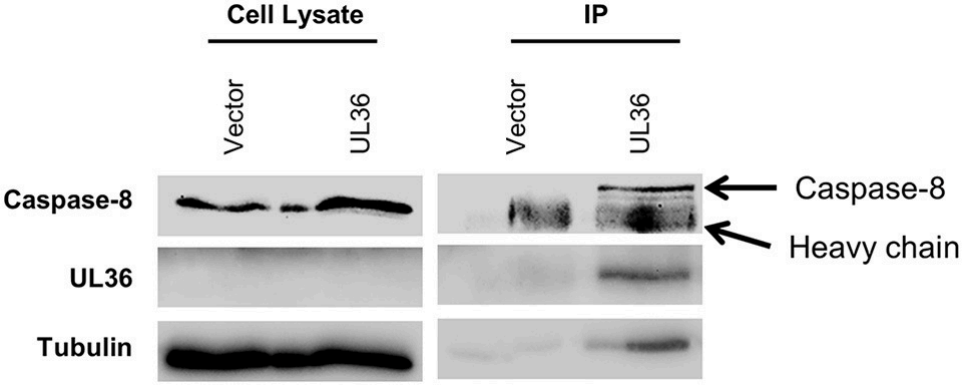
pUL36 forms complex with pro-caspase-8. Lysates from myc tagged UL36 or empty vector transfected cells were immunoprecipitated (IP) with the anti-myc antibody. Lanes designated IP are proteins bound with anti-myc antibody. Fifty-five kilo Daltons band of pro-caspase-8 was detected by immunoblot analysis using antibodies against murine caspase-8 1G12.

The pUL36 protein blocks the Fas-mediated apoptosis in human cells by binding to pro-caspase-8 and inhibiting its activation (17). Since the UL36 expression from the ΔM36.MCMV prevented apoptosis in infected murine macrophages, we tested if UL36 physically interacts with murine caspase-8. To determine this, co-immunoprecipitation assay was performed. For this purpose, NIH3T3 cells were transfected with myc-tagged UL36. Cell lysates were immunoprecipitated with anti-myc antibody coated beads and protein complexes were separated by SDS PAGE. Immunoblot analysis was performed using anti-caspase-8 and anti-UL36 antibodies. UL36 was detected in anti-myc immunoprecipitates from myc-tagged-UL36 transfected NIH3T3 cells, whereas it was not detected in control cells transfected with empty vector. Also, pro-caspase-8 was detected in the immunoprecipitates from UL36-transfected NIH3T3 murine cells but not from the controls, indicating that the protein pUL36 interacted with the murine pro-caspase-8 (Figure 7).

## Discussion

Cytomegalovirus is an extremely slowly growing virus, which necessitates antiapoptotic functions to protect the infected cell from cell-intrinsic apoptosis induced by metabolic stress (Goldmacher et al., 1999; Brune et al., 2003) but also from apoptosis triggered by extrinsic signaling (Skaletskaya et al., 2001; Mccormick et al., 2003, 2010; Menard et al., 2003; Ebermann et al., 2012). While previous work demonstrated that the MCMV gene M36 (Menard et al., 2003; Cicin-Sain et al., 2008) and the HCMV gene UL36 are required for viral growth in macrophages (Mccormick et al., 2010), and that both gene products target the same signaling pathway by binding to the highly conserved host target protein caspase-8 (Skaletskaya et al., 2001; Menard et al., 2003), it has remained unclear whether their function is interchangeable, and if UL36 would be functional in murine cells. We show here that UL36 may be detected along with caspase 8 in the enriched immunoprecipitates of the transfected cells, although its concentrations were too low to be detectable in whole cell lysates. Therefore, our results indicate binding a highly conserved molecular interaction between M36/UL36 proteins and murine/human caspase-8 targets.

Similar approaches were conducted to study the HCMV chemokine receptor (CKR) homologs UL33 and US28, or the HCMV protein kinase UL97. Farrell et al. developed ΔM33/UL33 and ΔM33/UL28 MCMV viruses, to replace the MCMV CKR homolog M33 with HCMV genes UL33 or UL28 (Farrell et al., 2011). Although, M33 and UL33 share homology, UL33 could not adequately complement the M33 gene and the recombinant virus suffered from *in vivo* growth defect. This hints that viral CKR homologs may be highly optimized to work in the host species that they co-evolved with. Similarly, UL97-MCMV was developed to study the UL97 protein, a protein kinase of HCMV that phosphorylates the antiviral drug ganciclovir, resulting in its targeted inhibition of DNA replication in HCMV-infected cells. UL97 protein partially substituted the M97 protein and rescued the growth deficit seen upon ΔM97.MCMV *in vivo* infection of mice (Wagner et al., 2000). In spite of the aforementioned caveats, these studies provide us with tools to study HCMV genes *in vivo*. Similarly, MCMV^UL36^ can be used to study the pUL36 (vICA) in the context of virus infection *in vivo*, because our experiments with MCMV^UL36^ demonstrated that UL36 functionally replaces the MCMV M36 gene, and largely rescues the growth defect shown by ΔM36.MCMV.

Zymosan activated macrophages have been shown to induce apoptosis in primary fibroblasts (MEFs) infected with ΔM36.MCMV, by secreting TNFα (Ebermann et al., 2012). However, it remains unclear how infection with ΔM36.MCMV, but not MCMV wild-type or MCMV^UL36^ induces apoptosis in the macrophage monocultures upon infection. One possible explanation might be that MCMV-infection deactivates macrophages, which release pro-apoptotic ligands that activate the FADD-mediated machinery and induce apoptosis in a paracrine fashion, but further studies are required to elucidate the underlying mechanism. The MCMV^UL36^ completely rescued the apoptosis in RAW264.7 and J774A.1, but the inhibition of apoptosis in the IC-21 cells was not complete. The incomplete inhibition of apoptosis, as compared to wild-type MCMV infection, might be explained by different affinity of binding of UL36 and M36 protein to the murine pro-caspase-8. Considering that the murine and human caspases have inherent differences in their sequences, where the homology between the human and the murine caspase-8 is 76% (Van De Craen et al., 1998), and that the sequence homology between M36 and UL36 is only 19% (Mccormick et al., 2003), it is reasonable to assume that the binding affinity of UL36 to the murine caspase-8 is different than that of M36. However, this aspect needs clarification by more detailed studies of M36 and UL36 binding to murine caspase-8.

We have previously shown that the growth of ΔM36.MCMV can be rescued by overexpression of a dominant-negative FADD variant (FADD^DN^) introduced ectopically in the MCMV genome and driven by the CMV i.e., promoter (Cicin-Sain et al., 2008). FADD^DN^ contains the death domain to associate with trimeric complex of death receptors, but lacks the N-terminal death effector domain required to activate caspase-8 (Chinnaiyan et al., 1995). Thus, it blocks caspase-8 mediated signaling and was able to compensate for the absence of M36 in the infected cell (Chinnaiyan et al., 1996). The *in vivo* fitness of the ΔM36.MCMV was not completely rescued by FADD^DN^ and ΔM36.FADD^DN^.MCMV showed moderate attenuation in some organs (Cicin-Sain et al., 2008). The attenuation of ΔM36.FADD^DN^.MCMV may either reflect differences in the molecular targets of M36 and FADD^DN^, or differences in expression kinetics, where FADD^DN^ is overexpressed by an exceedingly strong ectopic promotor. In term of expression kinetics, our current model differs from the previous one, because the homologous UL36 protein was expressed via the M36 promotor, and the splice acceptor and donor sites of M36 remained intact, arguing that splice efficiency of M36 is retained. This excludes any confounding factors associated with gene expression kinetics or amount, indicating that UL36 expression levels equal to that of M36 are enough to protect the infected cell from apoptosis and confer growth fitness. MCMV^UL36^ completely rescued the virus growth in liver and spleen. However, the UL36 expression only partially rescued the growth in the salivary glands. The low titers in the salivary glands might be either due to growth defects or to the inability of the virus to spread to distant organs from the initial site of infection. It has been shown that the M36 gene plays an important role in the dissemination of MCMV from the initial site of infection (Cicin-Sain et al., 2005) and monocytes are regarded as the vehicle for MCMV dissemination to distant tissues (Smith et al., 2004; Daley-Bauer et al., 2014). The monocytes do not support productive viral replication, but upon their migration into tissues they differentiate into macrophages, upon which productive virus replication initiates. The incomplete inhibition of apoptosis by MCMV^UL36^ (Figure 6) might therefore partly explain the poor *in vivo* spread and the incomplete rescue of virus replication in salivary glands. However, further experiments are required to validate this hypothesis.

Notwithstanding the moderate loss of fitness in salivary glands, the recombinant MCMV^UL36^ provides a novel tool to study the UL36 gene and demonstrates that UL36 may act as a determinant of viral fitness in the context of *in vivo* infection. In combination with previous studies, our data also suggest a vital role of the UL36 gene for *in vivo* fitness of HCMV (Skaletskaya et al., 2001; Mccormick et al., 2003, 2010). While antiviral substances targeting viral replication are available, these drugs exhibit toxic side-effects (e.g., suppression of hematopoiesis) and may result in escape mutations of the target genes (Ramanan and Razonable, 2013). The suggested vital role of the UL36 gene for viral *in vivo* fitness and the fact that M36 and UL36 exert their anti-apoptotic function by direct binding to pro caspase-8 (Skaletskaya et al., 2001; Menard et al., 2003), presents a novel antiviral target; targeting the anti-apoptotic function of UL36 could provide a new class of anti-viral compounds. As UL36 and M36 proteins engage identical molecular targets, the developed model can help studies of anti-viral compounds against the UL36 gene *in vivo*. The system will hence allow us to validate pharmacokinetics and toxicity properties of promising lead candidates targeting UL36 in the context of MCMV infection while additionally giving insight into their antiviral efficacy *in vivo*. This experimental model therefore, has a strong potential to support studies of human biology and applications in clinical medicine.

## Author contributions

MZC, BK, CP, MB, RC, and LB performed experiments. TL provided reagents. AS, SJ, IS, and LC designed the study. MZC and LC wrote the manuscript.

### Conflict of interest statement

The authors declare that the research was conducted in the absence of any commercial or financial relationships that could be construed as a potential conflict of interest.
