# Supplementary material for: UL36 Rescues Apoptosis Inhibition and In vivo Replication of a Chimeric MCMV Lacking the M36 Gene
Source: Front Cell Infect Microbiol. 2017 Jul 14;7:312. doi: 10.3389/fcimb.2017.00312 (PMC5509765; doi:10.3389/fcimb.2017.00312)
Supplement: Supplementary file 1 [file DataSheet1.docx]

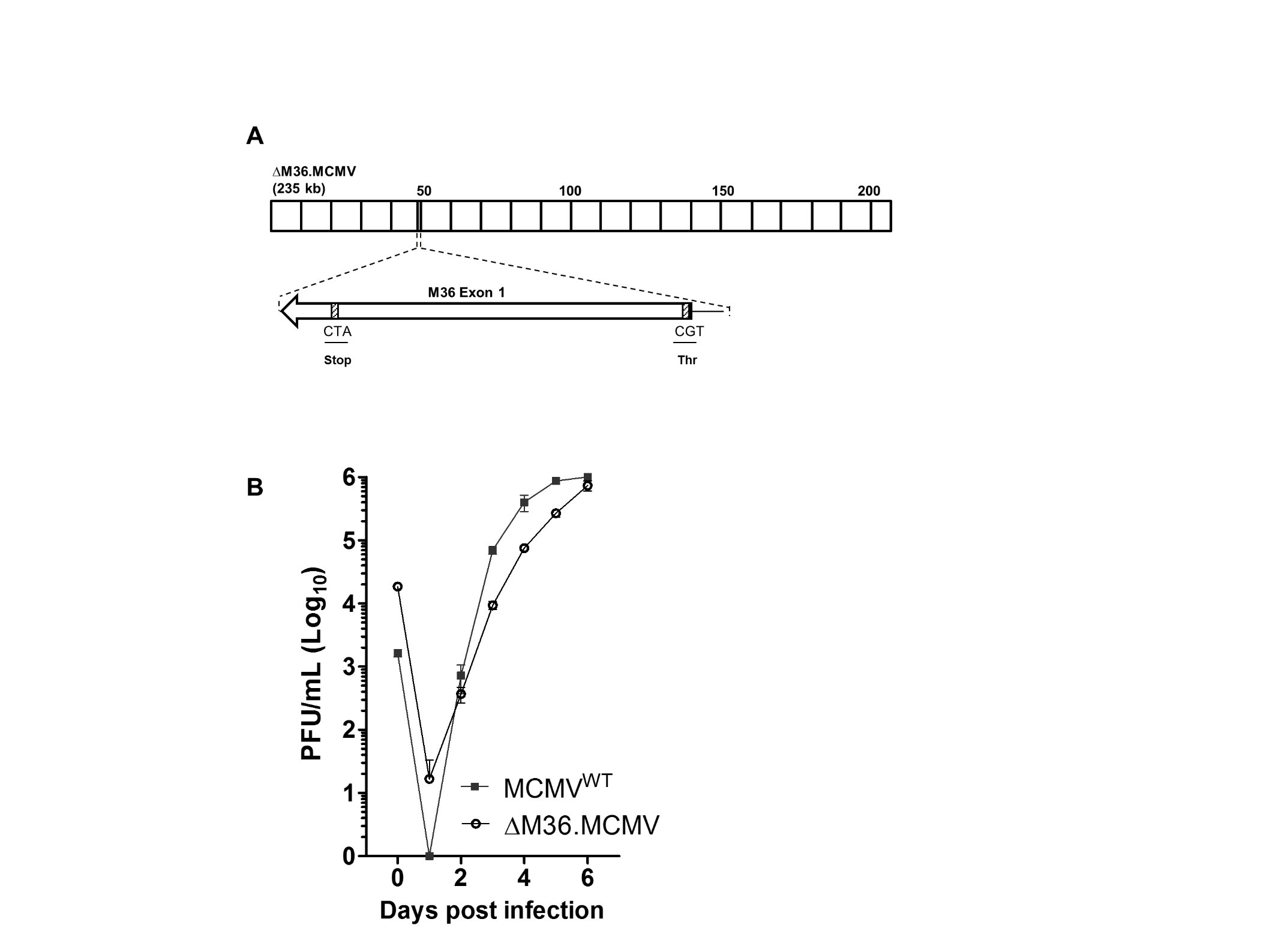


Figure S1. Recombinant ∆M36.MCMV generation A) The start codon and second methionine (ATG) codon of the M36 gene in MCMV genome were replaced with CGT and stop codon, respectively, using homologous En passant recombination. B) To test for general growth properties, of ∆M36.MCMV, NIH3T3 cells were infected with indicated viruses at an MOI of 0.1. The supernatants were collected at indicated days post infection and titrated on C57BL/6 MEFs. Values show the mean of three biological replicates and error bars represent SEM.
