# Supplementary figures and images for: UL36 Rescues Apoptosis Inhibition and In vivo Replication of a Chimeric MCMV Lacking the M36 Gene
Source: Front Cell Infect Microbiol. 2017 Jul 14;7:312. doi: 10.3389/fcimb.2017.00312 (PMC5509765; doi:10.3389/fcimb.2017.00312)

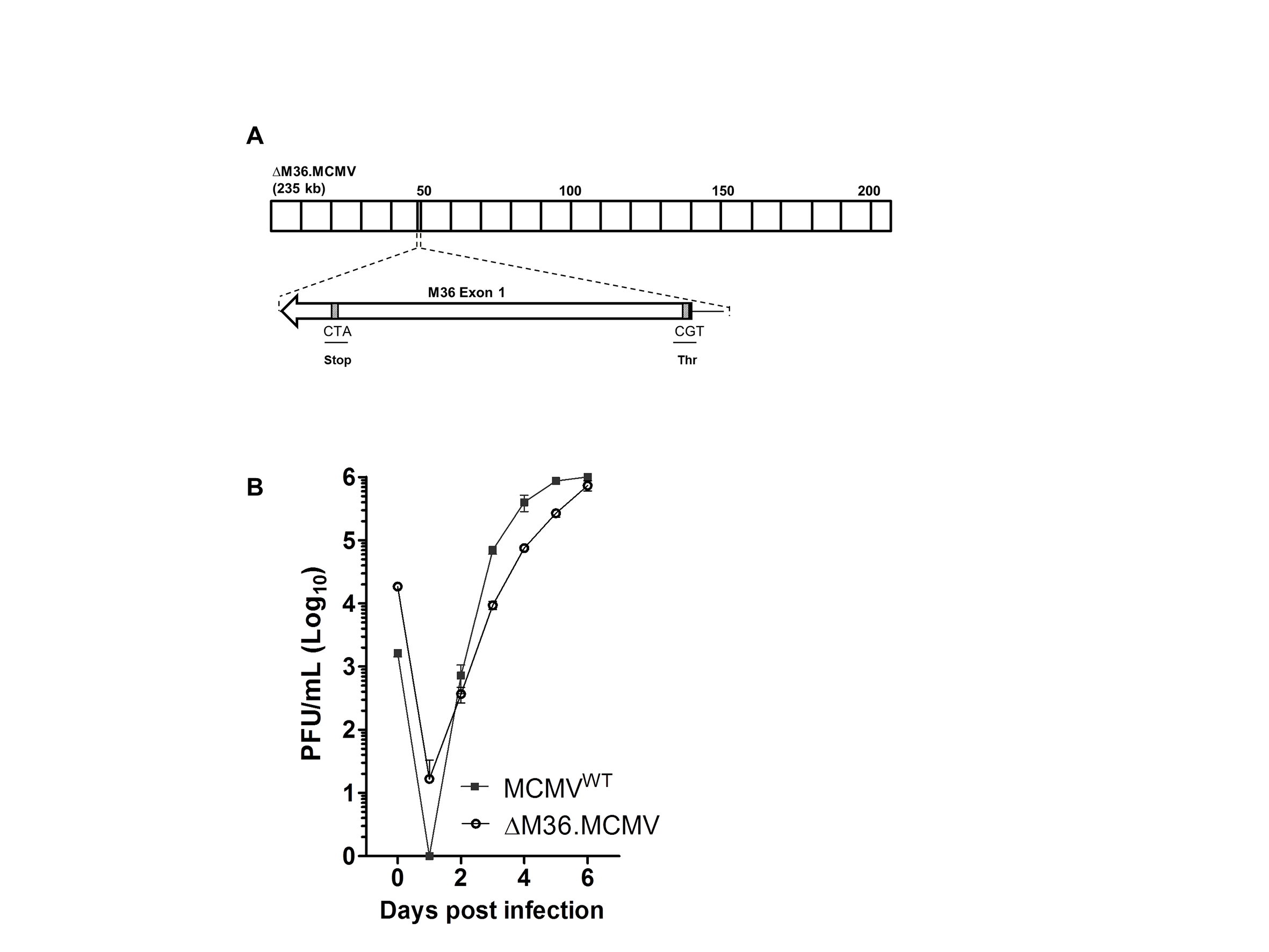

Supplement: Supplementary file 2 [file Image1.TIF]
